# Frequency-Regulated Repeated Micro-Vibration Promotes Osteoblast Differentiation Through BMP Signaling in MC3T3-E1 Cells

**DOI:** 10.3390/life15040588

**Published:** 2025-04-03

**Authors:** Ayumu Matsushita, Tada-aki Kudo, Kanako Tominami, Yohei Hayashi, Takuya Noguchi, Takakuni Tanaka, Satoshi Izumi, Keiko Gengyo-Ando, Atsushi Matsuzawa, Guang Hong, Junichi Nakai

**Affiliations:** 1 Division of Oral Physiology, Tohoku University Graduate School of Dentistry, Sendai 980-8575, Japan; matushita.ayumu.s6@dc.tohoku.ac.jp (A.M.); kanako.tominami.c2@tohoku.ac.jp (K.T.); satoshi.izumi.b8@tohoku.ac.jp (S.I.); keiko.ando.b2@tohoku.ac.jp (K.G.-A.); junichi.nakai.a5@tohoku.ac.jp (J.N.); 2 Cell Resource Center for Biomedical Research, Institute of Development, Aging and Cancer, Tohoku University, Sendai 980-8575, Japan; yohei.hayashi.e2@tohoku.ac.jp; 3 Graduate School of Life Sciences, Tohoku University, Sendai 980-8577, Japan; 4 Laboratory of Health Chemistry, Graduate School of Pharmaceutical Sciences, Tohoku University, Sendai 980-8578, Japan; takuya.noguchi.a7@tohoku.ac.jp (T.N.);; 5 Division for Globalization Initiative, Tohoku University Graduate School of Dentistry, Sendai 980-8575, Japan; tanat222@yahoo.co.jp (T.T.); hong.guang.d6@tohoku.ac.jp (G.H.)

**Keywords:** MC3T3-E1 cells, frequency-regulated repeated micro-vibration, osteoblast differentiation, bone morphogenetic protein, alkaline phosphatase

## Abstract

Physical stimulation, which is a key factor affecting the metabolism of osteoblasts and their precursor cells, plays an important role in bone remodeling; however, the role of micro-vibrations in osteoblast differentiation is unclear. In the present study, we determined the effects of frequency-regulated repeated micro-vibration (FRMV) on cell proliferation and established a method to induce osteoblast differentiation through FRMV using the mouse pre-osteoblast-like cell line MC3T3-E1, which is widely used in bone metabolism research. The results indicated that FRMV significantly influenced the proliferation of MC3T3-E1 cells in a normal growth medium. FRMV at 42.2 Hz significantly promoted proliferation, whereas FRMV at 92.1 Hz showed no effect on the proliferation rate. Moreover, FRMV at 42.2 Hz significantly increased alkaline phosphatase (ALP) enzyme activity and ALP gene expression in MC3T3-E1 cells. Treatment with LDN193189, a bone morphogenetic protein (BMP) signaling inhibitor, revealed that the FRMV-induced upregulation in ALP enzyme activity and ALP gene expression were significantly suppressed in MC3T3-E1 cells. The results suggest that the FRMV protocol developed in the present study induces osteoblast differentiation through the BMP signaling pathway. Thus, FRMV may contribute to the development of effective bone regeneration technologies.

## 1. Introduction

Osteoporosis is a skeletal disorder characterized by reduced bone strength and an increased risk of fractures. With an aging global population, it has become one of the most common non-communicable diseases [1,2,3]. Osteoporosis significantly affects mortality rates, daily living activities, and quality of life in both men and women over 50 years of age while also imposing a substantial economic burden on healthcare systems. In Japan, where the aging rate is the fastest and the elderly population is the highest, the incidence of fractures resulting from osteoporosis has been consistently rising, which has raised concerns about a similar impact in other rapidly aging East Asian countries [4]. Patients with osteoporosis are more susceptible to developing periodontal disease, and the severity of such conditions tends to be greater [5]. Conversely, tooth loss caused by periodontal disease reduces mastication ability, which in turn affects the digestion and absorption of nutrients. As a result, deficiencies in vitamin D and calcium, as well as malnutrition, may occur, potentially exacerbating osteoporosis [6]. Drug therapies for the prevention and treatment of osteoporosis can have serious side effects [7,8], which highlights the need for noninvasive and low-risk preventive and therapeutic methods. In this context, physical stimulation is considered a promising approach to improve these conditions [9,10,11,12].

Bone regeneration is regulated by the balance between bone formation and resorption, with osteoblasts playing a crucial role in the bone formation process. Osteoblasts are sensitive to physical stimulation, and ultrasound [13,14], electromagnetic fields [15,16], and thermal stimulation [17] promote osteoblast differentiation. Mechanical stimulation is also known to affect bone formation, as evidenced by the bone loss experienced by astronauts in microgravity [18] and the strengthening of bone through walking rehabilitation [19]. At the cellular level, microgravity [20] and compressive gas stress [21] cause cells to sense mechanical stimulation and respond morphologically and metabolically. In addition, external mechanical stresses, such as stretch stimulation [22,23], have been studied for their effects on the proliferation and differentiation of pre-osteoblasts into osteoblasts as well as on calcification. The combined use of electrical stimulation [24] and ultrasound stimulation [25] suggests a broad potential for clinical applications in both sports and medicine.

Studies suggest that mechanical vibration stimulation can heal fractures at the tissue level [26,27] The application of low-intensity, high-frequency whole-body vibration (WBV) in mice and rats promotes bone formation and the healing of damaged bone [28,29,30]; however, many aspects of the underlying mechanisms remain unclear [31]. In addition, there are reports of the promotion of osteoblast proliferation and differentiation at the cellular level using simple devices that apply mechanical vibration stimulation [32,33]. These studies suggest that both horizontal [34,35] and vertical [32,34,36,37] vibrational orientations relative to the cell adhesion surface affect cell proliferation and differentiation. Despite these findings, many aspects of the mechanisms of mechanical vibration stimulation at the cellular level remain unclear.

The bone morphogenetic protein (BMP) signaling pathway is an important pathway in mammalian bone formation [38,39]. When BMP binds to a complex composed of BMP type I and type II receptors, it initiates intracellular signal transduction via Smad proteins, which in turn induce the expression of downstream genes, such as alkaline phosphatase (ALP) [40]. BMP also activates non-Smad signaling pathways, including the mitogen-activated protein kinase pathway [41]. LDN193189 is a selective BMP signaling inhibitor that suppresses the kinase activity of BMP type I receptors, such as BMP receptor IA and BMP receptor IB [42].

In the present study, we hypothesized that a vibration load named frequency-regulated repeated micro-vibration (hereafter, FRMV), which uses a high-humidity-compatible culture cell micro-vibrator that allows for vibrating cells in a 5% CO_2_ incubator and also for the setting of an original micro-vibration (MV) control program, could induce osteoblast differentiation of pre-osteoblasts or pre-osteoblast-like cell lines through an unknown mechanism. To test this hypothesis, we used the mouse MC3T3-E1 cell line, a well-known osteoblast differentiation model for studying bone formation in vitro [43]. The MC3T3-E1 cell line was established in 1981 by Kodama and colleagues at the School of Dentistry, Ohu University. It is a pre-osteoblast-like cell line derived from the calvaria of neonatal mice [44,45] and is widely used to study the mechanisms of osteoblast differentiation in vitro. Under the above hypothesis, we examined the effects of FRMV with a constant frequency on osteoblast differentiation in MC3T3-E1 cells. Specifically, we determined the effect of FRMV on ALP enzyme activity and ALP gene expression level, markers of osteoblast differentiation. Moreover, we examined the role of the BMP signaling pathway at the early stages of FRMV-induced osteoblast differentiation using a pharmacologic inhibitor. Overall, we report a novel method for regulating osteoblast differentiation in MC3T3-E1 cells using FRMV and discuss the underlying biological mechanisms.

## 2. Materials and Methods

### 2.1. Cells and Reagents

The pre-osteoblast-like cell line MC3T3-E1 was obtained from the RIKEN BioResource Research Center (Tsukuba, Japan) and stored in liquid nitrogen (Saisan Co., Saitama, Japan). Penicillin/streptomycin and ALP yellow substrate solutions were purchased from Sigma-Aldrich (St. Louis, MO, USA). Dulbecco’s Modified Eagle Medium (DMEM), phosphate-buffered saline (PBS), Triton X-100, NaOH, glutamic acid, NaCl, glycine, and sucrose were obtained from Fujifilm Wako Pure Chemical Corporation (Tokyo, Japan). Tween 80 was purchased from MP Biomedicals (Solon, OH, USA). Recombinant human BMP2 (PeproTech, Rocky Hill, NJ, USA) was dissolved in LF6 buffer (5 mM glutamic acid, 5 mM NaCl, 2.5% glycine, 0.5% sucrose, and 0.01% Tween 80). LDN193189, which is a selective inhibitor of BMP type I receptors, was purchased from Cayman Chemical (Ann Arbor, MI, USA) and dissolved in dimethyl sulfoxide (Fujifilm Wako Pure Chemical). FetalClone III artificial serum was purchased from Cytiva (Logan, UT, USA).

### 2.2. Cells Culture

Cell culture of MC3T3-E1 cells was performed like described previously [43,46,47,48,49,50,51,52]. The cells were cultured under normal growth conditions using a growth medium of DMEM supplemented with 10% (*v*/*v*) FetalClone III artificial serum and penicillin/streptomycin solution. The cells were incubated at 37 °C in a 5% CO_2_ incubator and observed using a phase-contrast microscope, Leica DM IL LED (Leica Microsystems, Wetzlar, Germany).

### 2.3. FRMV Stimulation

**Figure 1:**
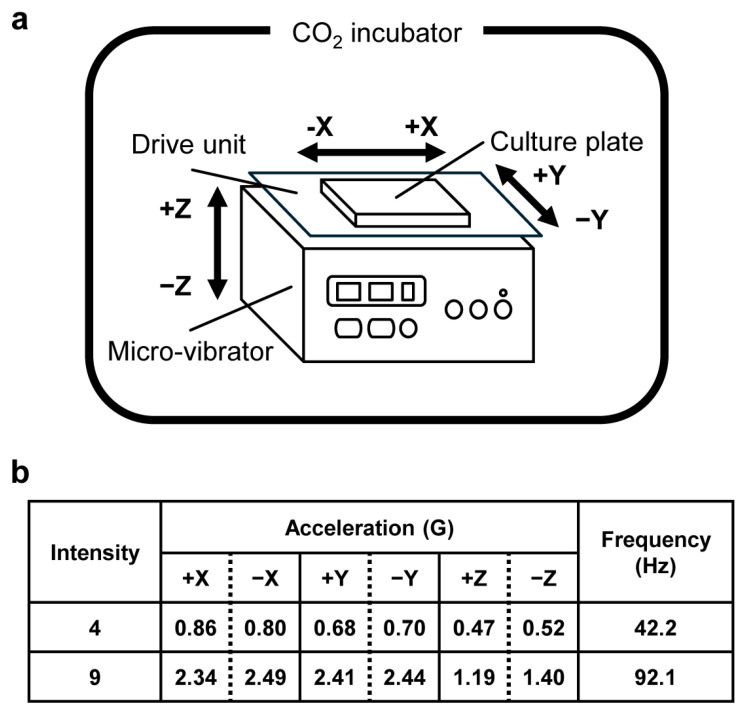
Schematic diagram of the FRMV stimulation experiment. (**a**) Culture plates seeded with cells were placed on a cell culture micro-vibrator (NSSB-300N, Nepa Gene). This micro-vibrator exhibited drip-proof and waterproof properties, and the cells were vibrated in a 5% CO_2_ incubator. (**b**) Three-dimensional movement of the micro-vibrator (based on the product specifications provided by Nepa Gene). FRMV, frequency-regulated repeated micro-vibration.

In the present study, a moisture- and drip-proof micro-vibrator (NSSB-300N, Nepa Gene, Chiba, Tokyo) was installed inside a 5% CO_2_ incubator to enable FRMV stimulation on MC3T3-E1 cells within the incubator. This micro-vibrator allows the vibration intensity to be set at nine levels, from the weakest at level 1 to the strongest at level 9, with standard specifications. It provides a consistent vibration frequency in the range of 14.2 Hz to 92.1 Hz, delivering micro-vibration (MV) stimulation to the cells triaxially: lateral (x), fore-aft (y), and vertical (z) axes (Figure 1). Culture plates seeded with cells were placed on the top surface of the vibration plate (drive unit) and subjected to stimulation according to the manufacturer’s instructions. For FRMV stimulation of MC3T3-E1 cells, two intensity settings were used: one with a vibration frequency of 42.2 Hz (intensity setting 4) and another with a vibration frequency of 92.1 Hz (intensity setting 9) (acceleration values are shown in Figure 1).

**Figure 2:**
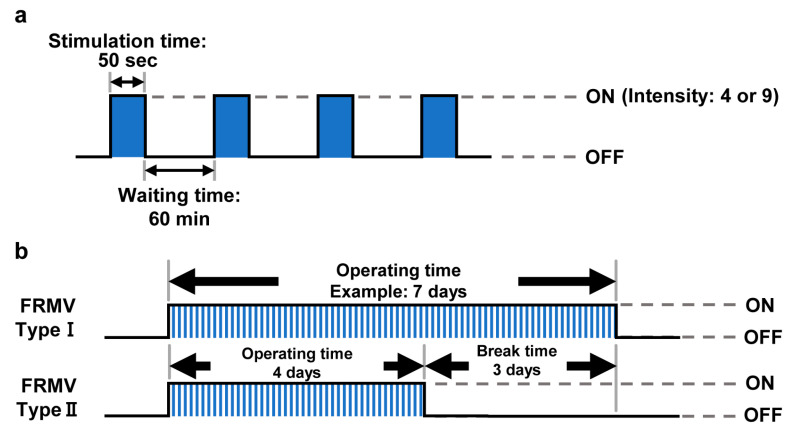
FRMV stimulation program. (**a**) Basic FRMV stimulation program. The program applied MV stimulation for 50 s per 1 h rest. (**b**) Schematic diagram of the FRMV treatment protocol in MC3T3-E1 cells. MC3T3-E1 cells received two types of FRMV stimulation using the basic program (**a**). For FRMV Type I, the cells were subjected to MV stimulation using the above basic program every day until the end of the intended stimulation period. For FRMV Type II, stimulation was applied for 4 days per 3-day break time. These procedures were repeated until the end of the intended stimulation period. FRMV, frequency-regulated repeated micro-vibration. MV, micro-vibration.

The stimulation time of vibration and the waiting time can also be preset on the micro-vibrator (NSSB-300N). Using this function, two FRMV programs were created to stimulate MC3T3-E1 cells. For the FRMV Type I program, the cells were repeatedly subjected to 50 s of MV stimulation with 1 h intervals daily until the end of the intended stimulation period (Figure 2a,b). For the FRMV Type II program, MV stimulation was repeatedly applied for 50 s with 1 h intervals during the first 4 days per each 3-day break time (Figure 2a,b).

### 2.4. Cell Proliferation Assay

MC3T3-E1 cells were seeded at a density of 2 × 10^3^ cells/well in a 6- or 24-well culture plate (TPP, Trasadingen, Switzerland) with growth medium and cultured under normal growth conditions for 3 days with or without FRMV stimulation. Cell counts were quantified on days 0, 1, 2, and 3 following FRMV stimulation, as described previously [53]. Briefly, the cells were observed using a phase-contrast microscope (Leica DM IL LED, Leica Microsystems), and three random fields from each well were captured using an MC120 HD digital camera (Leica Microsystems). The number of adherent cells in each captured field was counted, and each data point represents the counts obtained from 3 to 4 independent wells. The effects of FRMV on cell proliferation of MC3T3-E1 cells under normal growth conditions for 3 days were also examined using a hemocytometer (NanoEntek, Seoul, South Korea) in accordance with the manufacturer’s protocol.

### 2.5. Osteoblast Differentiation Induced by FRMV

To establish an efficient method for inducing osteoblast differentiation through FRMV, MC3T3-E1 cells were seeded into a 24-well culture plate (TPP) at a density of 2 × 10^4^ or 3 × 10^4^ cells per well. To acquire low-proliferation conditions by contact inhibition of the cells, the cells were then cultured with growth medium for 2–3 days until they reached 100% confluence, upon which the medium was replaced with a differentiation-inducing medium (a low-serum medium compared with the growth medium used in the present study: DMEM containing 5% (*v*/*v*) FetalClone III artificial medium and penicillin/streptomycin solution). This was considered day 0 of the experiment, and FRMV stimulation was initiated.

Using one of the two FRMV programs (FRMV Type I or Type II) described above, FRMV stimulation was applied to the cells for up to 21 days. The degree of osteoblast differentiation was assessed by measuring the enzymatic activity of ALP derived from MC3T3-E1 cells using an ALP enzyme activity assay. For the control experiment, a group of cells without FRMV stimulation was used as the negative control, whereas a group treated with BMP2 (10 ng/mL), a tentative inducer of osteoblast differentiation in MC3T3-E1 cells, was used as the positive control. The effects of FRMV on cell proliferation of MC3T3-E1 cells during osteoblast differentiation were also evaluated using a hemocytometer (NanoEntek) in accordance with the manufacturer’s protocol.

### 2.6. Alkaline Phosphatase (ALP) Enzyme Activity Assay

An ALP enzyme activity assay was performed as described previously [43] using cell samples during differentiation to evaluate osteoblast differentiation in MC3T3-E1 cells induced by external stimulation. After inducing cell differentiation for 7, 14, or 21 days with or without FRMV, the cells were washed three times with PBS, and 200 μL ALP yellow (pNPP) substrate solution were added. Next, 2 μL of 10% Triton X-100/PBS were added to each well, and the cells were incubated at 37 °C for 15 min. After the solution turned yellow, the reaction was stopped by adding 75 μL of 2 M NaOH. The absorbance was measured at 450 nm using a microplate spectrophotometer, Infinite M200 (Tecan, Männedorf, Switzerland). This assay was performed in quadruplicate.

### 2.7. Quantitative Real-Time Polymerase Chain Reaction (QRT-PCR)

RNA was extracted using the RNeasy Mini Kit (Qiagen, Hilden, Germany) and the QIA shredder column (Qiagen) based on the manufacturer’s instructions. First-strand cDNA was synthesized from the extracted total RNA using SuperScript III reverse transcriptase (Thermo Fisher Scientific, Waltham, MA, USA) or ReverTra Ace qPCR RT kit (Toyobo, Osaka, Japan) with random primers (Promega, Madison, WI, USA). QRT-PCR was performed using Power SYBR Green Master Mix (Thermo Fisher Scientific) based on previous reports [54,55]. The primers for the ALP and β-actin genes were purchased from Takara Bio (Shiga, Japan) (Table 1). The PCR signals were detected following the manufacturer’s instructions for the CFX Connect (Bio-Rad, Berkeley, CA, USA). Expression data were normalized to β-actin or glyceraldehyde 3-phosphate dehydrogenase (GAPDH) levels, which served as the internal control.

### 2.8. Statistical Analysis

All data are presented as the mean ± standard deviation. Statistical analysis was performed with the statistical package JSTAT v22.0J for Windows (Sato, Japan). Significant differences between groups were identified by two-tailed Student’s *t*-tests or one- or two-way analysis of variance followed by Tukey’s test, as appropriate. *p*-values < 0.05 were considered statistically significant.

## 3. Results

### 3.1. Effects of FRMV on Cell Proliferation Under Normal Growth Conditions

To determine the effect of FRMV (for details, see Materials and Methods) on the viability of MC3T3-E1 cells, we examined the effects of FRMV Type I (Figure 2) using the following vibration intensities: intensity level 4 and maximum intensity level 9 (Figure 1b). MC3T3-E1 cells under normal growth conditions (see Materials and Methods for details) were cultured in growth medium for 3 days with or without the FRMV application. Using a microscope, the number of adherent cells on the bottom of each well in the culture plate was determined on days 0, 1, 2, and 3 after the start of vibration.

**Figure 3:**
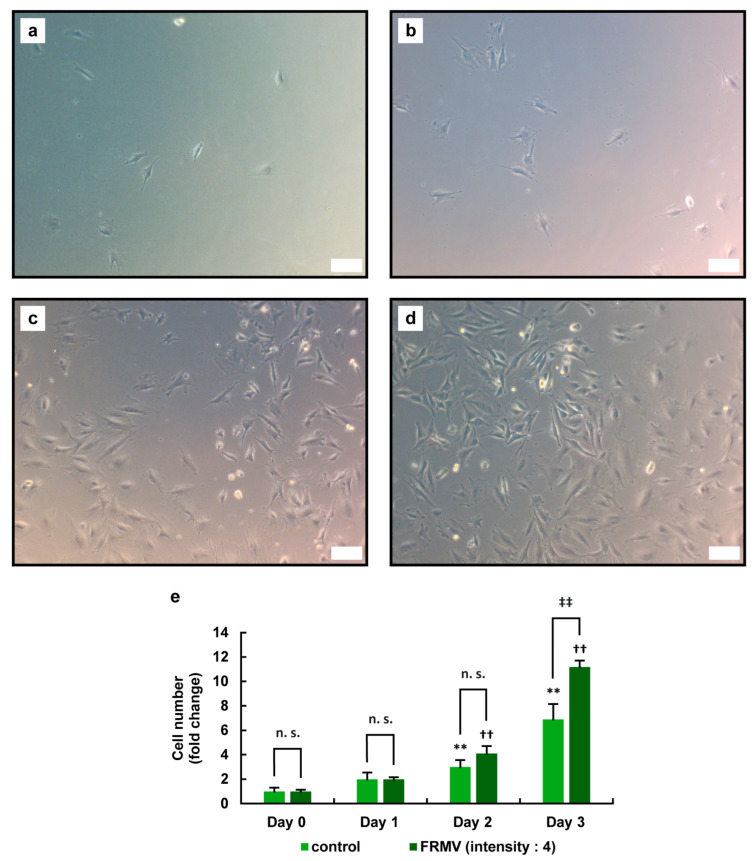
Effects of FRMV at stimulation intensity 4 on the proliferation of MC3T3-E1 cells. MC3T3-E1 cells were cultured in growth medium for 1 day in a 24-well culture plate and subjected to FRMV Type I stimulation (a repeating program of 50 s of vibration stimulation per 1 h of rest each day) at intensity level 4 or cultured without FRMV treatment for 3 days. During the culture period, phase-contrast microscopy images were captured on days 0, 1, 2, and 3 after the initiation of FRMV treatment. Phase-contrast images of the cells on day 0 without FRMV exposure (**a**,**b**) and on day 3 in the absence (**c**) or presence (**d**) of FRMV are shown. Scale bars: 100 µm. (**e**) The number of cells attached to the bottom of the plate was measured using the captured phase-contrast microscope images. The data represent the mean ± standard deviation obtained from three independent experiments. ** *p* < 0.01 indicates a significant difference compared with the day 0 control without FRMV stimulation. †† *p* < 0.01 indicates a significant difference compared with the day 0 control with FRMV stimulation. ‡‡ *p* < 0.01. n.s., not significant. FRMV, frequency-regulated repeated micro-vibration.

**Figure 4:**
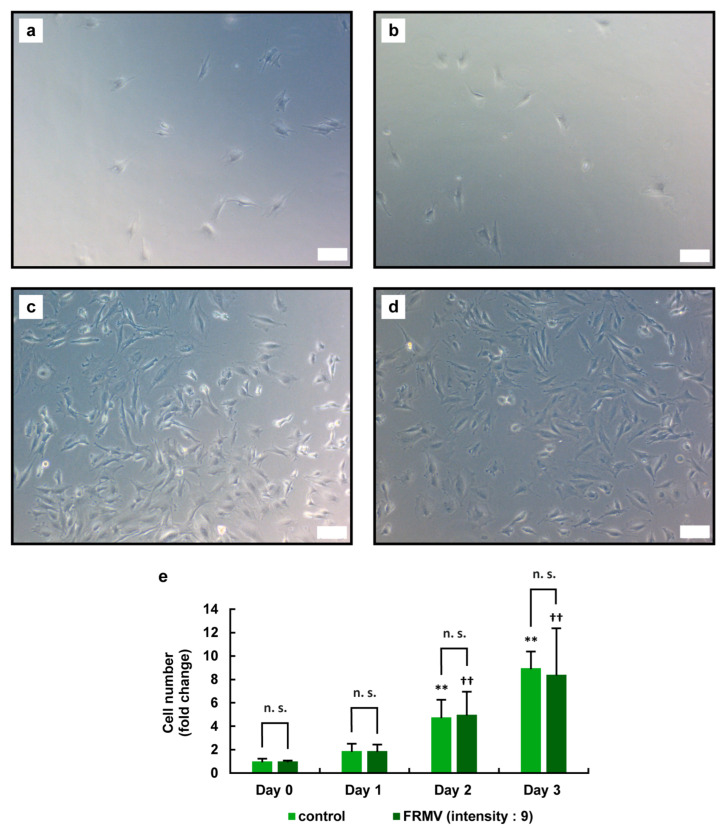
Effects of FRMV at stimulation intensity 9 on the proliferation of MC3T3-E1 cells. MC3T3-E1 cells were cultured in growth medium for 1 day in a 24-well culture plate and subjected to FRMV Type I stimulation (a repeating program of 50 s of vibration stimulation per 1 h of rest each day) at intensity level 4 or cultured without FRMV treatment for 3 days. During the culture period, phase-contrast microscopy images were captured on days 0, 1, 2, and 3 after the initiation of FRMV treatment. Phase-contrast images of the cells on day 0 without FRMV exposure (**a**,**b**), on day 3 in the absence (**c**) or presence (**d**) of FRMV are shown. Scale bars: 100 µm. (**e**) The number of cells attached to the bottom of the plate was measured using the captured phase-contrast microscope images. The data represent the mean ± standard deviation obtained from three independent experiments. ** *p* < 0.01 indicates a significant difference compared with the day 0 control without FRMV stimulation. †† *p* < 0.01 indicates a significant difference compared with the day 0 control with FRMV stimulation. n.s., not significant. FRMV, frequency-regulated repeated micro-vibration.

On day 3 after the initiation of vibration stimulation, the number of MC3T3-E1 cells under FRMV with intensity level 4 was significantly increased compared with that in the control group without FRMV (Figure 3). In contrast, while the number of MC3T3-E1 cells increased from day 0 to day 3 at intensity level 9, no significant changes were observed in the number of the cells compared with that in the control group without FRMV (Figure 4).

**Figure 5:**
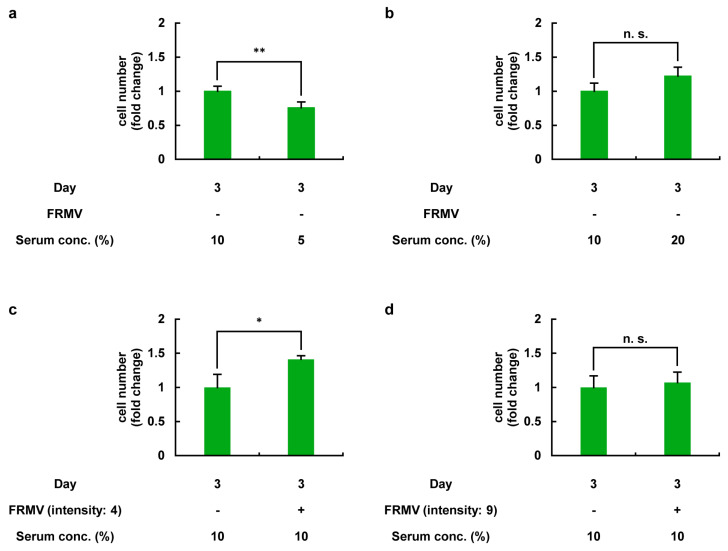
Effects of FRMV at stimulation intensities 4 and 9 on the proliferation of MC3T3-E1 cells. MC3T3-E1 cells were initially cultured in normal growth medium containing 10% serum for one day in a six-well culture plate. After replacing the medium with fresh medium containing 5%, 10%, or 20% serum, the cells were cultured with or without FRMV Type I stimulation (a repeating program of 50 s of vibration stimulation followed by 1 h of rest per day) at intensity 4 or 9 for three days. On day 3, cell numbers under each culture condition were examined using a hemocytometer. The numbers of cells cultured in growth medium containing 5% (**a**) or 20% (**b**) serum on day 3 in the absence of FRMV exposure are shown. The numbers of cells cultured in growth medium containing 10% serum on day 3 in the presence of FRMV at stimulation intensity 4 (**c**) or 9 (**d**) are also shown. The data represent the mean ± standard deviation obtained from three independent experiments. * *p* < 0.05. ** *p* < 0.01. n.s., not significant. FRMV, frequency-regulated repeated micro-vibration. Serum conc., serum concentration.

To confirm the above results, the effects of FRMV at stimulation intensity levels 4 and 9 on the proliferation of MC3T3-E1 cells were evaluated using a hemocytometer (Figure 5). In this experiment, MC3T3-E1 cells were cultured in a growth medium containing the indicated serum concentrations (5%, 10%, or 20%), with or without FRMV Type I stimulation (a repeating program of 50 s of vibration stimulation followed by 1 h of rest each day) for three days. On day 3, cell numbers under each culture condition were examined using a hemocytometer.

On day 3, the number of MC3T3-E1 cells cultured in a growth medium containing 5% serum, in the absence of FRMV stimulation, was significantly lower than that in the control group, as expected, which used a medium containing 10% serum (Figure 5a). In contrast, while the number of MC3T3-E1 cells cultured in a growth medium containing 20% serum without FRMV stimulation tended to increase, no significant difference was observed compared to the control group using a medium with 10% serum (Figure 5b). Moreover, under normal growth conditions using a normal growth medium containing 10% serum, the number of MC3T3-E1 cells subjected to FRMV stimulation at intensity 4 was significantly higher than that in the control group without FRMV (Figure 5c), consistent with the results shown in Figure 3. In contrast, regarding the number of MC3T3-E1 cells on day 3 under FRMV stimulation at intensity 9, no significant difference was observed compared to the control group without FRMV (Figure 5d), consistent with the results shown in Figure 4.

### 3.2. Effect of FRMV on ALP Enzyme Activity

**Figure 6:**
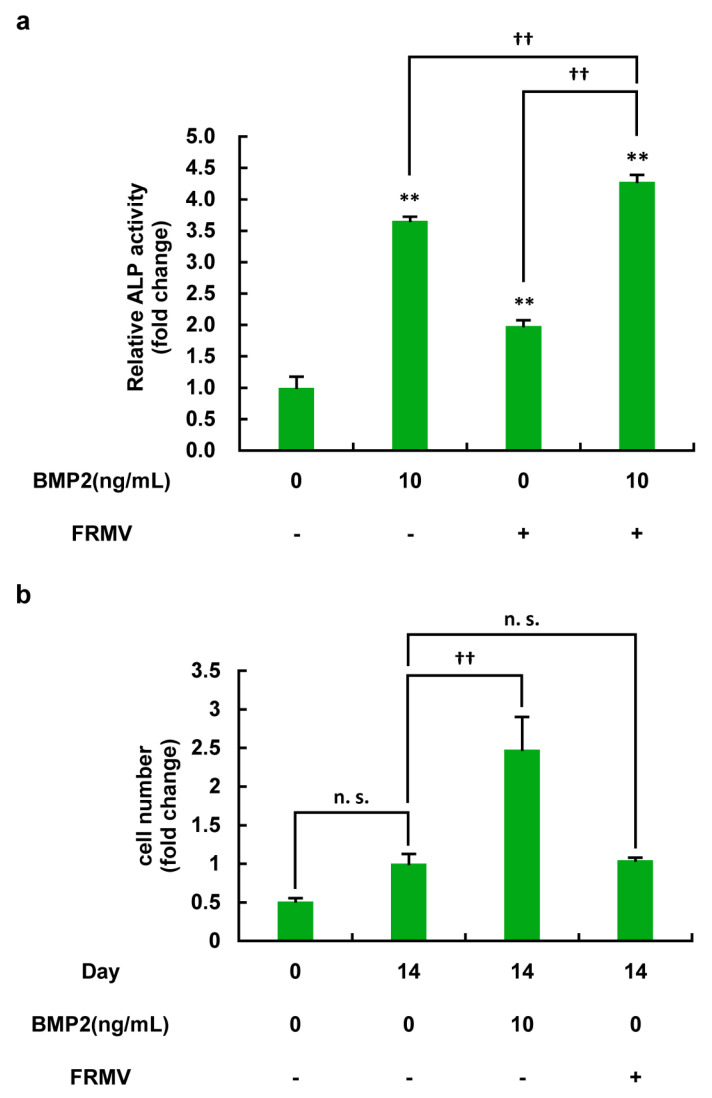
Effects of FRMV on ALP activity and cell proliferation in MC3T3-E1 cells under a low-serum condition. One hundred percent confluent MC3T3-E1 cells were cultured in a 24-well culture plate using a differentiation-inducing medium, placed on a vibration device, and FRMV Type I (a repeating program that applies 50 s of vibration stimulation per 1 h of rest each day) at vibration intensity level 4 was applied for 14 days with or without BMP2 (10 ng/mL). (**a**) On day 14, ALP activity was measured for each condition to assess the degree of osteoblast differentiation. BMP2 was used as a positive control for inducing osteoblast differentiation and increasing ALP enzyme activity. The data represent the mean ± standard deviation obtained from four independent experiments. ** *p* < 0.01 indicates a significant difference compared with the day 14 control without FRMV stimulation. †† *p* < 0.01. (**b**) On day 0 and day 14, cell numbers were counted in each condition to evaluate cell proliferation under the low-serum condition optimized for osteoblast differentiation of the cells. BMP2 was used as a positive control for inducing osteoblast differentiation. The data represent the mean ± standard deviation obtained from four independent experiments. †† *p* < 0.01. n.s., not significant. BMP, bone morphogenetic protein. ALP, alkaline phosphatase. FRMV, frequency-regulated repeated micro-vibration.

To determine the effect of FRMV on the osteoblast differentiation of MC3T3-E1 cells, we applied FRMV Type I at vibration intensity level 4. MC3T3-E1 cells under low-proliferation conditions (see Materials and Methods for details) were exposed for 14 days in the presence or absence of BMP2 (10 ng/mL), and ALP enzyme activity and cell number were measured. As shown in Figure 6a, BMP stimulation, which serves as a positive control for osteoblast differentiation, significantly increased ALP enzyme activity. Similar to BMP2, FRMV stimulation alone also significantly increased ALP enzyme activity. Moreover, when FRMV and BMP2 treatment were combined, ALP enzyme activity was significantly higher compared with either one alone. On the other hand, as shown in Figure 6b, the FRMV had no significant effect on cell number, at least on day 14 compared with the day 14 control without FRMV stimulation in the cells, whereas BMP stimulation significantly increased the cell number on day 14 compared with the day 14 control without FRMV stimulation.

### 3.3. Time-Course Evaluation of FRMV-Dependent ALP Enzyme Activity Changes

**Figure 7:**
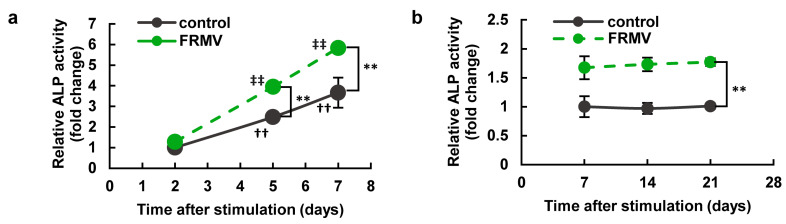
Time course of the increase in ALP activity in MC3T3-E1 cells induced by FRMV. (**a**,**b**) One hundred percent confluent MC3T3-E1 cells were cultured in a 24-well culture plate using a differentiation-inducing medium and incubated for (**a**) 2, 5, or 7 days or (**b**) 7, 14, or 21 days in the presence or absence of the FRMV Type I program (a repeating program that applied 50 s of vibration stimulation per 1 h of rest each day) at vibration intensity level 4. ALP enzyme activity was measured at each time point to evaluate the degree of osteoblast differentiation. The data represent the mean ± standard deviation obtained from four independent experiments. †† *p* < 0.01 indicates a significant difference compared with the day 2 control without FRMV stimulation. ‡‡ *p* < 0.01 indicates a significant difference compared with the day 2 control with FRMV stimulation. ** *p* < 0.01. ALP, alkaline phosphatase. FRMV, frequency-regulated repeated micro-vibration.

A time course of FRMV-dependent changes in ALP enzyme activity was evaluated. MC3T3-E1 cells were cultured with or without FRMV Type I stimulation at vibration intensity level 4 for up to 21 days, and ALP enzyme activity was assessed on days 2, 5, 7, 14, and 21 after the initiation of vibration. As shown in Figure 7, the time-dependent upregulation of ALP enzyme activity was observed in both the negative control group (without FRMV) and the FRMV-stimulated group until day 7. Compared with the negative control group, FRMV significantly increased ALP enzyme activity on days 5 and 7 after the start of vibration. Moreover, the increased ALP enzyme activity induced by FRMV remained significantly higher compared with that of the negative control group from day 7 to day 21.

### 3.4. Comparison of the Effects of the Two FRMV Programs on the ALP Enzyme Activity

**Figure 8:**
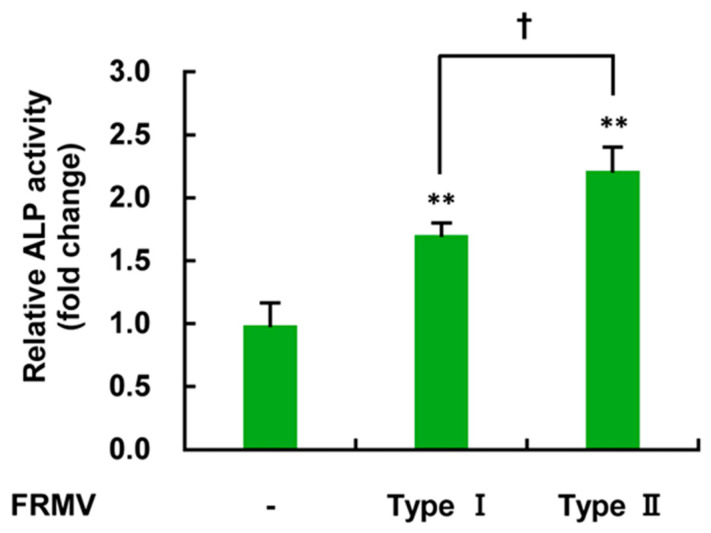
Comparison of the degree of FRMV-dependent upregulation of ALP enzyme activity using two FRMV programs. One hundred percent confluent MC3T3-E1 cells cultured in a 24-well culture plate with differentiation-inducing medium were subjected to two FRMV programs (FRMV Type I and FRMV Type II) at vibration intensity level 4 for 14 days or were cultured for 14 days without FRMV treatment. ALP enzyme activity at each time was measured to determine the degree of osteoblast differentiation. The data represent the mean ± standard deviation of four independent experiments. ** *p* < 0.01 indicates a significant difference compared with the control without FRMV stimulation. † *p* < 0.05. ALP, alkaline phosphatase. FRMV, frequency-regulated repeated micro-vibration.

To determine the effect of the differences in FRMV stimulation at vibration intensity level 4 on increased ALP enzyme activity, MC3T3-E1 cells under low-proliferation conditions were exposed to FRMV Type I and Type II (Figure 2b) for 14 days, and ALP enzyme activity was measured. As shown in Figure 8, FRMV Type I and II significantly increased ALP activity in MC3T3-E1 cells on day 14 compared with that in the control group without FRMV stimulation. Unexpectedly, MC3T3-E1 cells subjected to FRMV Type II, which had a shorter total vibration stimulation duration, exhibited significantly higher ALP enzyme activity on day 14 compared with cells subjected to FRMV Type I.

### 3.5. Inhibition of FRMV-Dependent Upregulation of ALP Enzyme Activity by LDN193189

**Figure 9:**
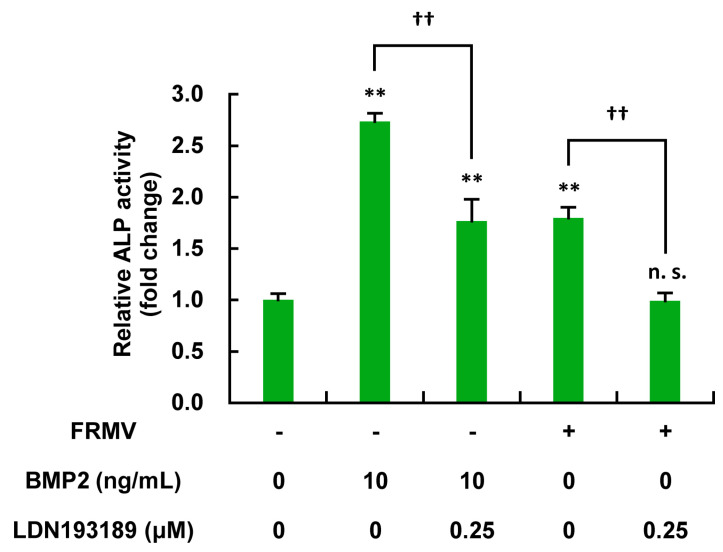
Inhibition of FRMV-dependent upregulation of ALP enzyme activity in MC3T3-E1 cells. One hundred percent confluent MC3T3-E1 cells were cultured in a 24-well culture plate with differentiation-inducing medium and exposed to FRMV Type I (which repeatedly applied 50 s of vibration stimulation per every 1 h of rest) at vibration intensity level 4 or BMP2 (10 ng/mL) as a positive control, in the presence or absence of LDN193189 (0.25 μM) for 14 days. ALP enzyme activity under each condition was measured to determine the degree of osteoblast differentiation. The data represent the mean ± standard deviation of four independent experiments. ** *p* < 0.01 indicates a significant difference compared with the control without FRMV stimulation. †† *p* < 0.01. n.s., not significant. ALP, alkaline phosphatase. BMP, bone morphogenetic protein. FRMV, frequency-regulated repeated micro-vibration.

To determine whether the BMP signaling pathway is involved in the FRMV-dependent upregulation of ALP enzyme activity, the effects of the BMP signaling inhibitor LDN193189 were assessed on the osteoblast differentiation of MC3T3-E1 cells induced by FRMV or BMP2 as a control. Specifically, MC3T3-E1 cells under low-proliferation conditions were exposed to FRMV Type I at vibration intensity level 4 or BMP2 (10 ng/mL) for 14 days in the presence or absence of LDN193189 (0.25 µM), and ALP enzyme activity was measured. As shown in Figure 9, LDN193189 treatment significantly inhibited the FRMV-dependent upregulation of ALP activity observed in MC3T3-E1 cells following FRMV stimulation, which was similar to the effects observed with BMP2. This suggests that the BMP signaling pathway is involved in the upregulation of FRMV-dependent ALP activity.

### 3.6. Induction of ALP Gene Expression by FRMV

#### 3.6.1. BMP2-Dependent Upregulation of ALP Gene Expression in MC3T3-E1 Cells and Its Inhibition by LDN193189

**Figure 10:**
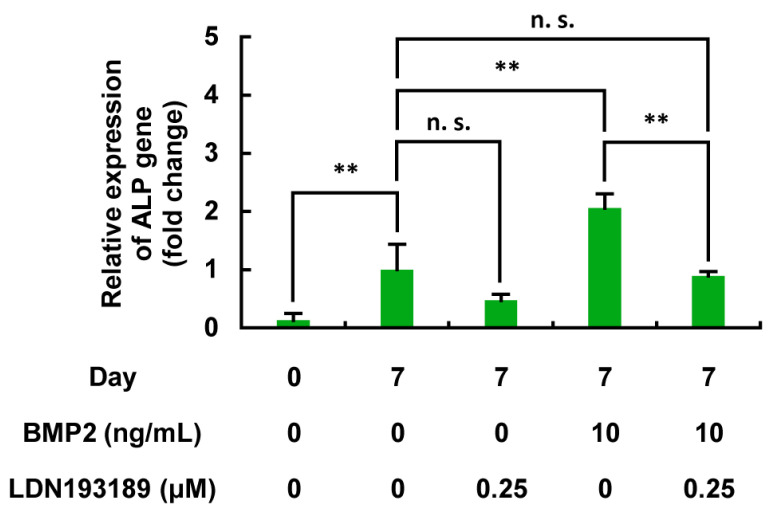
Effects of LDN193189 on the BMP2-dependent upregulation of ALP gene expression in MC3T3-E1 cells. One hundred percent confluent MC3T3-E1 cells were subjected to BMP2 (10 ng/mL) stimulation for 7 days in the presence or absence of LDN193189 (0.25 μM). The expression of ALP mRNA was normalized based on that of the β-actin gene as an endogenous control. The results are presented as the relative changes compared with the day 7 control. The data represent the mean ± standard deviation from four independent experiments. ** *p* < 0.01. n.s., not significant. ALP, alkaline phosphatase. BMP, bone morphogenetic protein.

The results presented in Figure 6, Figure 7, Figure 8 and Figure 9 prompted us to determine the mechanism underlying the FRMV-dependent upregulation of ALP enzyme activity per cell in MC3T3-E1 cells. For this purpose, we next investigated the effect of FRMV on the ALP gene expression during osteoblast differentiation of the cells. Firstly, MC3T3-E1 cells under low-proliferation conditions were stimulated with BMP2 as a positive control, and the expression of the ALP gene, a tentative differentiation gene marker of osteoblast differentiation, was measured on day 7 after stimulation. BMP2 (10 ng/mL) significantly increased the expression of the ALP gene on day 7 after stimulation (Figure 10). Furthermore, when the effect of the BMP signaling inhibitor LDN193189 on the BMP-dependent upregulation of ALP gene expression was examined, LDN193189 treatment significantly suppressed the upregulation of ALP gene expression induced by BMP2, whereas LDN193189 alone showed no significant effects on the ALP gene expression compared to the day 7 control without BMP stimulation.

#### 3.6.2. FRMV-Dependent Upregulation of ALP Gene Expression in MC3T3-E1 Cells and Its Inhibition by LDN193189

**Figure 11:**
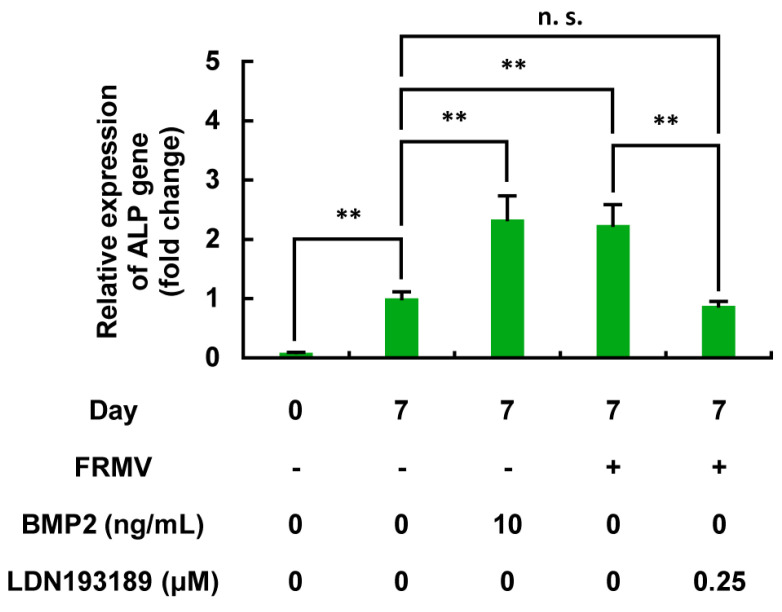
Effects of LDN193189 on the FRMV-dependent upregulation of ALP gene expression in MC3T3-E1 cells. One hundred percent confluent MC3T3-E1 cells were subjected to FRMV Type I stimulation at vibration intensity level 4 for 7 days in the presence or absence of LDN193189 (0.25 μM). BMP2 (10 ng/mL) was used as a positive control. The expression of ALP mRNA was normalized based on that of the GAPDH gene as an endogenous control. The results are presented as the relative changes compared with the day 7 control. The data represent the mean ± standard deviation from four independent experiments. ** *p* < 0.01. n.s., not significant. ALP, alkaline phosphatase. BMP, bone morphogenetic protein. FRMV, frequency-regulated repeated micro-vibration. GAPDH, glyceraldehyde 3-phosphate dehydrogenase.

Next, FRMV Type I at vibration intensity level 4 was applied to the MC3T3-E1 cells under low-proliferation conditions, and the expression of the ALP gene was measured on day 7 after stimulation. FRMV significantly increased the expression of the ALP gene on day 7 after the start of stimulation, which was similar to the control with BMP2 (Figure 11). Furthermore, when the effect of the BMP signaling inhibitor LDN193189 on FRMV-dependent upregulation of ALP gene expression was examined, LDN193189 treatment significantly suppressed the upregulation of ALP gene expression induced by FRMV, similar to the effect observed with BMP2.

## 4. Discussion

The present study clarified a novel effect of FRMV on the proliferation and differentiation of MC3T3-E1 cells and partially elucidated its mechanism of action. Specifically, FRMV significantly increased or had no effects on the cell proliferation rate of MC3T3-E1 cells under normal growth conditions, which was dependent on the FRMV settings without the need for additional differentiation-inducing factors. As we hypothesized, we also observed a significant FRMV-induced upregulation of ALP enzyme activity (an indicator of osteoblast differentiation) per cell under low proliferation conditions without enhancing cell proliferation. Furthermore, FRMV upregulates the expression of the ALP gene (a gene marker of osteoblast differentiation) in MC3T3-E1 cells, which contributes to the upregulation of ALP enzyme activity. Moreover, the BMP signaling inhibitor LDN193189 significantly suppressed the upregulation of ALP gene expression and enzyme activity induced by FRMV.

We used MC3T3-E1 cells, a model of osteoblast differentiation, because osteoblasts participate directly not only in bone formation but also in the differentiation and activation of osteoclasts and thus play an essential role in bone remodeling. There are several unique molecular characteristics of osteoblasts. First, they exhibit high ALP enzyme activity. They also synthesize high levels of type I collagen as well as noncollagenous proteins, such as osteopontin and osteocalcin. Moreover, they can form calcified material and express receptors for estrogen, active vitamin D, and parathyroid hormone. They also produce cytokines, such as BMP, transforming growth factor beta (TGF-β), and insulin-like growth factor (IGF) [56]. In the present study, we focused on ALP enzyme activity and the ALP gene as differentiation markers to evaluate osteoblast differentiation induced by FRMV; however, it will be necessary to further determine how other osteoblast characteristics, such as type I collagen and osteocalcin, change in response to FRMV in future studies.

ALP increases the local concentration of phosphate at the onset of calcification and removes calcification inhibitors, such as pyrophosphate and adenosine triphosphate (ATP); thus, ALP is a marker of calcification. In addition, the ALP gene expression is increased during the early and mid-stages of osteoblast differentiation, which renders it useful for objectively evaluating osteoblast differentiation. Therefore, the ALP gene is a representative marker for osteoblast differentiation induction [43,57].

In previous studies on the vibration stimulation of osteoblasts, it was revealed that vibration stimulation can induce the proliferation and differentiation of osteoblasts; however, the characteristics of the experimental environments and conditions vary widely, and many aspects of the effects and appropriate use conditions of vibration stimulation remain unclear. The FRMV used in the present study was characterized by MV stimulation triaxially: lateral (x), fore-aft (y), and vertical (z) axes (Figure 1). In previous studies, the effect of vibration stimulation on osteoblast differentiation was reported using only horizontal [34,35] or only vertical [32,34,36,37] stimulation relative to the cell adhesion surface. The results from these studies using horizontal or vertical vibration stimulation indicate that vibration stimulation at frequencies of 30 Hz or 100 Hz in combination with the acceleration amplitude of 0.15 G or 1 G increased the ALP enzyme activity of mesenchymal stem cells. The highest increase was observed at 0.15 G and 100 Hz. Furthermore, higher ALP enzyme activity levels were observed for horizontal vibration stimulation compared with vertical stimulation under certain conditions [34]. Although vertical vibration stimulation increases the expression of the ALP gene in osteoblasts, particularly at 0.5 G and 50 Hz, no significant increase was observed at frequencies above 100 Hz [32].

Because the human body is generally exposed to three-dimensional vibrations, we hypothesized that the input and output should be considered simultaneously for all three axes from a clinical perspective because of the transmission characteristics of vibration in the body. WBV, which is already used in physical therapy and expanding into the medical field, is an example in which devices capable of applying vibration stimulation triaxially are used [58]. It affects bone formation; however, many aspects, such as the optimal vibration conditions and their effects on the bones, remain unclear. Therefore, we used MV stimulation triaxially to determine the effect of FRMV on osteoblast differentiation in an environment closer to a clinical setting.

In the present study, to elucidate an efficient method for inducing osteoblastic differentiation of MC3T3-E1 cells using FRMV, it is essential to first examine whether FRMV at various intensity is harmful or harmless to cell survival. Therefore, we first observed and examined the effects of FRMV on cell proliferation under normal growth conditions. In this context, since the micro-vibration device used in the present study allowed stimulation intensity to be set between 1 and 9, we first examined the effects of FRMV on cell proliferation using stimulation intensity 9, the maximum intensity, and stimulation intensity 4, which is approximately half of the maximum intensity. As a result, the following points became clear. FRMV at stimulation intensity 4 promoted the proliferation of MC3T3-E1 cells (Figure 3 and Figure 5c), whereas FRMV at stimulation intensity 9 showed no effects on the proliferation rate of MC3T3-E1 cells (Figure 4 and Figure 5d). The results suggest that the former FRMV conditions are not cytotoxic and exert a cell proliferation-promoting effect under normal culture conditions. Furthermore, FRMV may regulate the proliferation rate of pre-osteoblasts in a manner that depends on the frequency and acceleration of the FRMV.

To determine the effect of FRMV on osteoblast differentiation in MC3T3-E1 cells, we first confirmed that ALP enzyme activity was significantly increased after 14 days of BMP stimulation, which served as the positive control. When FRMV Type I at stimulation intensity 4 was applied to MC3T3-E1 cells under low-proliferation conditions for 14 days, FRMV stimulation alone also significantly increased ALP enzyme activity, similar to that following BMP stimulation. Furthermore, when FRMV and BMP stimulation were applied to the cells simultaneously, ALP enzyme activity significantly increased compared with each stimulation alone (Figure 6a). In this context, we also clearly showed that FRMV had no effects on cell proliferation during FRMV-induced osteoblast differentiation under low proliferation conditions (Figure 6b). This suggests that the observed FRMV-dependent upregulation of ALP enzyme activity was due to an increase in total ALP enzyme activity per cell. Additionally, we unexpectedly found that BMP2 can upregulate the cell proliferation rate of MC3T3-E1 cells even under low-proliferation conditions, characterized by low serum and contact inhibition. This finding highlights the need for further investigation to elucidate the mechanism underlying the observed differences in cell proliferation between BMP2 and FRMV under low proliferation conditions.

Regardless of the presence of FRMV stimulation, a time-course evaluation of ALP enzyme activity during FRMV Type I stimulation revealed a gradual increase in ALP enzyme activity from day 2 to day 7, which was maintained from day 7 to day 21. However, from day 5 onwards, FRMV Type I stimulation resulted in significantly higher ALP enzyme activity levels compared with the negative control group without FRMV stimulation (Figure 7). Moreover, FRMV Type I stimulation alone significantly increased ALP gene expression on day 7 (Figure 11). These results suggest that under low-proliferation conditions, FRMV alone can promote osteoblast differentiation, continuously enhancing ALP enzyme activity during the FRMV stimulation period, at least in part, through an unknown mechanism that can induce FRMV-dependent ALP gene upregulation. They also indicate that FRMV can promote BMP-dependent osteoblast differentiation.

To determine the effect of different FRMV stimulation times on FRMV-dependent upregulation of ALP enzyme activity, we applied two FRMV programs, FRMV Type I and Type II, at stimulation intensity 4 (Figure 2b). We treated MC3T3-E1 cells with each FRMV program under low-proliferation conditions for 14 days and measured ALP enzyme activity. Stimulation with both FRMV Type I and Type II significantly increased ALP enzyme activity compared with the control group, which was not subjected to FRMV loading. Unexpectedly, however, the group subjected to FRMV Type II, which had a shorter total vibration stimulation duration, exhibited significantly higher ALP enzyme activity compared with the FRMV Type I group (Figure 8). This suggests that the upregulation of ALP enzyme activity in MC3T3-E1 cells does not simply correlate with the FRMV stimulation duration; however, further studies of the FRMV stimulation program are needed.

We measured ALP enzyme activity in MC3T3-E1 cells exposed to BMP2 (10 ng/mL) or FRMV Type I stimulation at stimulation intensity 4 for 14 days under low-proliferation conditions, either in the presence or absence of LDN193189 (0.25 µM), which is a tentative BMP signaling inhibitor. As shown in Figure 11, LDN193189 treatment significantly suppressed the FRMV-dependent upregulation of ALP enzyme activity, similar to the case of BMP2. Moreover, under the same conditions, we found that the upregulation of ALP gene expression observed on day 7 after FRMV Type I stimulation at intensity 4 was also significantly suppressed by LDN193189 treatment, similar to that observed with BMP2 (Figure 10 and Figure 11). The results suggest that the upregulation of FRMV-dependent ALP enzyme activity requires the BMP signaling pathway through an unknown mechanism(s).

For gene expression analysis, β-actin is commonly used as a housekeeping gene for normalization [59]. However, as a cytoskeletal protein, actin production and formation can be readily influenced by mechanical forces [60], potentially affecting cell morphology. Therefore, using β-actin as a housekeeping gene may not be appropriate when MC3T3-E1 cells are stimulated with mechanical forces [61,62]. To address this, GAPDH was used instead to normalize ALP gene expression in cells stimulated with FRMV [25,63], as shown in Figure 11.

In the present study, as FRMV, MV stimulation lasting a few dozen seconds was repeatedly applied to MC3T3-E1 cells with rest periods. A similar stimulation protocol was previously reported in studies aimed at promoting cytoplasmic maturation in oocytes [64,65,66]. To our best knowledge, however, no previous reports have applied FRMV stimulation as described in the present study to assess proliferation or differentiation in MC3T3-E1 cells. Previous studies performed vibration stimulation for promoting osteoblast differentiation in MC3T3-E1 cells, in which MV with acceleration of less than 1 G and a frequency ranging from 1 Hz to 100 Hz was employed [67]. While the vibration methods used in those studies are not identical to FRMV, they share partial similarities. Previous studies involving the MV indicate that periodic MV stimulation with an acceleration amplitude of 0.3 G and a frequency of 40 Hz for 30 min per day promotes the proliferation, adhesion, and osteogenic differentiation of bone marrow mesenchymal stem cells and osteoblasts [67,68]. In addition, under the same conditions, ALP enzyme activity in MC3T3-E1 cells was significantly increased compared with non-vibrating conditions [69].

Other studies have described a type of vibration stimulation, primarily with an acceleration below 1 G and a frequency ranging from 20 Hz to 90 Hz, as low-magnitude high-frequency vibration (LMHFV) [70]. Studies on LMHFV have examined the effects of LMHFV on fracture healing in rats and humans [71,72,73], on bone remodeling in rats induced by LMHFV and WBV [28,30], and on bone mass in long-term bedridden individuals induced using LMHFV and WBV [74]. At the cellular level, LMHFV has also been evaluated in osteoblast differentiation studies using MC3T3-E1 cells [33,70,75,76,77,78]. The duration of vibration stimulation in LMHFV varies. For example, ALP enzyme activity was significantly decreased immediately after applying LMHFV at 0.49 G and 40 Hz for 30 min, compared with non-vibrating conditions [70]. In contrast, when LMHFV at 40 Hz was applied for 30 min per day for 3 days, the highest ALP enzyme activity was observed at 0.49 G among six different acceleration amplitudes (0.06, 0.14, 0.32, 0.49, 0.66, or 0.8 G) [37,78]. In addition, when LMHFV at 0.25 G and 35 Hz was applied for 20 min a day for 9 days, ALP enzyme activity on day 9 was significantly higher compared with non-vibrating conditions [77].

These reports also suggest that various phenomena may be triggered by different parameters of vibration stimulation (frequency, acceleration, and stimulation time). Therefore, a detailed comparative analysis using these different vibrational stimulation methods is necessary to further elucidate the molecular mechanisms of FRMV-dependent osteoblast differentiation induction.

In the present study, we used a waterproof vibration device amenable to high-humidity environments that enables the application of vibration stimulation inside a CO_2_ cell culture incubator. This successfully eliminated the need to take the cells out of the incubator each time FRMV stimulation is applied, thus avoiding temperature fluctuations from the outside air and pH changes that occur when cells are exposed to external conditions. Thus, this allows for a more accurate evaluation of the effects of vibration stimulation alone. Studies using vibration stimulation on various cell types within CO_2_ incubators have been described [32,64,65,66,67]. Therefore, the use of a highly water-resistant vibration device within an incubator is essential for our study, in which FRMV, a short MV stimulation applied for 50 s at 60-min intervals, was used to repeatedly stimulate cells.

## 5. Conclusions

In conclusion, we clarified that osteoblast differentiation, which is important in the bone formation process, was promoted by FRMV. Our results also suggest that similar to BMP2, FRMV requires the BMP signaling pathway to induce osteoblast differentiation and promote matrix synthesis in MC3T3-E1 cells. This highlights the need for further studies into the mechanism of action of FRMV and the identification of key signaling molecules that regulate FRMV-dependent osteoblast differentiation in MC3T3-E1 cells. Overall, our findings suggest that FRMV promotes the induction of osteoblast differentiation via the BMP signaling pathway. Further studies on FRMV may lead to the development of effective and useful bone regeneration techniques in the future.

## Figures and Tables

**Table 1:** Primer sequences were used for the quantitative real-time polymerase chain reaction (QRT-PCR).

GenBank Accession Number	Gene Name	Forward Primer (5′–3′)	Reverse Primer (5′–3′)
NM_007431.3	ALP	AATTGAATCGGAACAACCTGACTG	CCTCATGATGTCCGTGGTCAA
NM_007393.5	*β*-actin	CATCCGTAAAGACCTCTATGCCAAC	ATGGAGCCACCGATCCACA
NM_001411840.1	GAPDH	AGAGCAACAGGGTGGTGGAC	TGGGATAGGGCCTCTCTTGCT

ALP, alkaline phosphatase. GAPDH, glyceraldehyde 3-phosphate dehydrogenase.

## Data Availability

The original contributions presented in this study are included in the article. Further inquiries can be directed to the corresponding author.
